# Implementation of a Dynamic Co-Culture Model Abated Silver Nanoparticle Interactions and Nanotoxicological Outcomes In Vitro

**DOI:** 10.3390/nano11071807

**Published:** 2021-07-12

**Authors:** Nicholas J. Braun, Rachel M. Galaska, Maggie E. Jewett, Kristen A. Krupa

**Affiliations:** 1 Molecular Bioeffects Branch, Human Effectiveness Directorate, Wright Patterson Air Force Base, Dayton, OH 45433, USA; nickbraun9@gmail.com; 2 Department of Chemical and Materials Engineering, University of Dayton, Dayton, OH 45469-0256, USA; galaskar1@udayton.edu (R.M.G.); jewettm3@udayton.edu (M.E.J.)

**Keywords:** silver nanoparticle, nanotoxicology, cellular co-culture, dynamic flow, reactive oxygen species, cytokine production

## Abstract

The incorporation of engineered nanoparticles (NPs) into everyday consumer goods, products, and applications has given rise to the field of nanotoxicology, which evaluates the safety of NPs within biological environments. The unique physicochemical properties of NPs have made this an insurmountable challenge, as their reactivity and variable behavior have given rise to discrepancies between standard cell-based in vitro and animal in vivo models. In this study, enhanced in vitro models were generated that retained the advantages of traditional cell cultures, but incorporated the modifications of (1) inclusion of an activated immune element and (2) the presence of physiologically-relevant dynamic flow. Following verification that the human alveolar epithelial and macrophage (A549/U937) co-culture could be successfully sustained under both static and dynamic conditions, these cultures, in addition to a standard A549 static model, were challenged with 10 nm citrate coated silver NPs (AgNPs). This work identified a reshaping of the AgNP-cellular interface and differential biological responses following exposure. The presence of dynamic flow modified cellular morphology and reduced AgNP deposition by approximately 20% over the static exposure environments. Cellular toxicity and stress endpoints, including reactive oxygen species, heat shock protein 70, and secretion of pro-inflammatory cytokines, were found to vary as a function of both cellular composition and flow conditions; with activated macrophages and fluid flow both mitigating the severity of AgNP-dependent bioeffects. This work highlights the possibility of enhanced in vitro systems to assess the safety of engineered NPs and demonstrates their effectiveness in elucidating novel NP-cellular interactions and toxicological profiles.

## 1. Introduction

Due to their distinctive physicochemical properties, nanoparticles (NPs) have been incorporated into hundreds of consumer, medical, industrial, and military products and applications. Properties that make NPs advantageous for novel functions over their bulk counterparts include: increased reactivity, augmented transport potential, superior strength to weight ratios, distinctive optical properties, and heightened electric potential [1]. Of all the elemental compositions, silver nanoparticles (AgNPs) have been employed at greater rates than any other material [2]. Due to their robust antibacterial and antimicrobial capabilities, AgNPs have been incorporated into household appliances, bandages, textiles, bioremediation agents, and as a surface coating for electronics and medical devices [3,4]. Owing to their plasmonic properties and high conductivity, AgNPs are integrated into biological sensors, medical diagnostics, drug delivery vehicles, and photocatalysis platforms [5].

However, one considerable drawback to the widespread and commercial use of AgNPs is the significant health concerns that may arise as these particles, in particular, are associated with a strong toxicological profile. The toxicity responses identified following AgNP exposure are vast and include cell death, activation of several oxidative stress pathways, modification of basal signal transduction functionality, DNA damage, induction of inflammatory reactions, and modifications to genetic regulation [6,7,8]. Ionic dissolution, or the breakdown of the particles into silver ions, has been identified as a contributing factor to AgNP-dependent cytotoxicity, but it is not the sole mechanism of action for these particles [9,10]. The released ions freely translocate throughout a cellular system and disrupt crucial pathways by reacting with other soluble biomolecules, leading to the induction of apoptosis. 

Primary particle size is a predominant factor in AgNP-mediated cellular death, with smaller particles associated with greater rates of internalization and cytotoxicity [11]. Previous studies have determined that particles in the 10 nm or smaller range are the most toxic, as they are able to enter a cell through both direct diffusion and traditional forms of endocytosis [11,12]. In recent years it has become clear that the nano-cellular interface is a critical facet of nanotoxicology, as rates of particle deposition and interaction are driving forces for cellular consequences post exposure [13,14]. The physicochemical properties of NPs, including size, morphology, surface chemistry, agglomeration tendencies, and porosity, all influence the extent of particle interactions with the surrounding cellular system, and as such regulate NP-induced bioresponses [15,16]. Therefore, a critical step in NP safety evaluations is to assess the deposition efficiency, which is the percentage of NPs that are internalized or bound by the cellular system following exposure.

To date, the lion’s share of nanomaterial safety and health evaluations are carried out using in vitro systems, due to their flexibility, ease of use, rapid throughput, and reduced cost [17]. However, there exists a significant translation gap between cell based in vitro and animal in vivo responses following NP exposure; highlighting a current roadblock facing the field of nanotoxicology [18,19]. The likely cause for these model based discrepancies is the foundational difference in structure and behavior of in vitro versus in vivo models. For example, traditional in vitro systems are two dimensional, comprised of a single cell model, static, and contain no immune element, whereas in vivo models are three dimensional, multi-cellular by nature, dynamic, and possess a complex immune system to protect the body [20].

In order to overcome this challenge, scientists and engineers have begun modifying in vitro models to incorporate features that make cell based systems more physiologically relevant. For example, cells are being grown in scaffolds to allow both cells and NPs the ability to migrate in three dimensions, which has resulted in differential deposition and internalization rates [21,22]. Moreover, in order to recreate the influence of the cardiovascular system, studies have been completed that incorporate fluid dynamics into NP exposure systems. These studies have determined that fluid movement modified particle transport by adding a third lateral force, in addition to NP diffusion and sedimentation [23]. Altered dosimetry profiles within dynamic conditions converted into differential NP-dependent bioresponses, aligning with changes to the nano-cellular interface [24,25]. Lastly, to provide a level of protection from foreign material, such as NPs, initial studies have been carried out that incorporated representative immune elements [26]. These efforts have determined that the presence of immune cells has the potential to reduce NP-toxicity by seeking out and destroying particles and releasing pro-inflammatory cytokines which influence the behavior of surrounding cells [27,28].

This is one of the first studies in which two physiologically-relevant modifications were incorporated into an experimental in vitro system. The base was A549 human alveolar cells, which were selected owing to their extensive use in nanotoxicological investigations [29]. The activated macrophage line, U937, was then added to the system to create an immune-containing co-culture model for AgNP safety investigations. This co-culture was maintained under both static and dynamic conditions, allowing for investigation into the influence of fluid movement on the nano-cellular interface and resultant bioeffects. Citrate coated 10 nm AgNPs were implemented in this work, as small particles have been correlated to higher deposition and toxicity rates [30]. The presented results demonstrate that both executed in vitro modifications altered the nano-cellular interface and toxicological profile, highlighting the need to create in vitro systems of greater biological relevance.

## 2. Materials and Methods

### 2.1. In Vitro Cell Cultures

The human alveolar epithelial, A549, and the human monocytic, U937, cell lines were purchased from American Type Culture Collection (Manassas, VA, USA) and maintained in RPMI 1640 supplemented with 10% fetal bovine serum and 1% penicillin/streptomycin. The A549s were cultured in tissue culture treated dishes and sub-cultured every few days as necessary. The U937s were grown in suspension in T-flasks and subcultured every 3–4 days. A lung co-culture was utilized in this study, which consisted of A549 and differentiated U937 cells cultured together in a 3:1 ratio as previously described [27]. In order to differentiate the U937 monocytes into activated macrophages, the cells were seeded into a tissue culture treated flask and stimulated with 100 ng/mL of phorbol-12-myristate-13-acetate (PMA) for 48 h. Following differentiation, the U937s and A549s were removed from their individual culture flasks via trypsin, counted, and plated together at a 3:1 ratio at predetermined cell densities. All cell culture reagents were obtained from ThermoFisher (Waltham, MA, USA).

### 2.2. Dynamic Flow Setup

In order to establish a dynamic exposure environment, a multi-channel peristaltic pump (Ismatec, model #ISM939D; Cole Parmer, Vernon Hills, IL, USA) was implemented, as previously described [24,25]. The tubing, 1/16 inch inner diameter, was tightly secured into pre-drilled holes in individual wells of a 24-well plate, generating unilateral flow across the cellular surface. Prior to experimentation, the pump was primed to ensure that media levels were equivalent for both static and dynamic exposures. The entire pump system was placed inside a temperature controlled incubator to ensure no thermal fluctuations. The pump ran at a predetermined volumetric flow rate in order to produce a tube-side linear velocity of 0.2 cm/s, matching known capillary rates in vivo [31].

### 2.3. AgNP Characterization

10 nm, citrate coated AgNPs were purchased from nanoComposix (San Diego, CA, USA) in concentrated liquid form. The AgNP stock was stored in the dark at 4 °C, in accordance with the manufacturer’s recommendations. In order to confirm spherical morphology and determine primary particle size, transmission electron microscopy (TEM) analysis was completed on a Hitachi H-7600 (Tokyo, Japan). For all the remaining characterization assessments, the AgNPs were diluted to 25 µg/mL, in either water or media. The agglomerate size of the AgNPs, in both fluids, was carried out using an Anton Paar Litesizer 500 (Graz, Austria) dynamic light scattering machine. The zeta potential, which is a measure of the particle surface charge, was also determined using the Anton Paar Litesizer 500. The unique spectral signature for the AgNPs was visualized through ultraviolet–visible (UV–Vis) spectroscopy using a Varian Cary 5000 (Aligient; Sanata Clara, CA, USA). Lastly, in order to determine the extent of AgNP ionic dissolution, the particles were incubated in media or water and stored at 37 °C for a duration of 24 h. The AgNPs were then removed from solution via centrifugation at 10,000 rpm for 15 min, after which the ion containing supernatant underwent Ag+ analysis through inductively coupled plasma mass spectrometry (ICP-MS; Perkin Elmer NexION 300D; Hebron, KY, USA).

### 2.4. Evaluation of the Nano-Cellular Interface

Cellular morphology and nano-interactions were visualized via fluorescent imaging. Cells were seeded onto a chambered slide at a density of 2 × 10^5^ cells/side, with the co-culture at a 3:1 A549:U937 ratio, and incubated overnight. The next day the cells were washed and the chambers were replenished with either fresh media or a 5 µg/mL AgNP solution, under the denoted conditions. After a 24 h incubation, the cells were washed, fixed with 4% paraformaldehyde, blocked with bovine serum albumin, and incubated with Alexa Fluor 555-phalloidin and DAPI stains for actin and nuclear targeting, respectively (ThermoFisher). The cells were then visualized using an Olympus BX41 microscope (Aetos Technologies; Auburn, AL, USA), with the AgNPs imaged using a Cytoviva ultraresolution attachment.

For deposition efficiency, the denoted cell models were seeded into 24-well plates at a density of 2 × 10^5^ cells/well and allowed to adhere overnight. The next day 5 µg/mL AgNP was introduced to the cell cultures and incubated under static or dynamic conditions for a duration of 24 h. At the end of the exposure period, the AgNP-containing media was removed, the cells were washed extensively to remove any unbound AgNPs, collected, and the cellular containing or bound silver content was quantified via ICP-MS analysis.

### 2.5. Cellular Viability Assessments

In this study, mammalian cell viability was determined via two assays, MTS which monitors active mitochondrial function, and lactate dehydrogenase (LDH) quantification which is released after membrane damage [32]. For both metrics, cells were plated into 24-well plates at a density of 2 × 10^5^ cells/well. The next day the cells were washed and replenished with either fresh media or a 5 µg/mL AgNP solution, under the denoted exposure conditions. After a 24 h incubation viability was determined using the MTS or CytoTox 96 Non-Radioactive Cytotoxicity (LDH) assay kits (Promega; Madison, WI, USA), in accordance with the manufacturer’s instructions. For LDH, a positive toxicity control, in which all the cells were lysed, was used to determine the degree of cytotoxicity. In both cases, untreated A549 cells served as the control for normalization.

### 2.6. Intracellular Reactive Oxygen Species

Intracellular stress was determined via reactive oxygen species (ROS) levels following AgNP exposure and for basal stress assessment. The cellular systems were seeded in 24-well plates at 2 × 10^5^ cells per well and incubated overnight. The next day, the cells were washed and incubated with the DCFH-DA probe (Thermo Fisher Scientific) for 30 min. After staining, the cells were again washed, dosed with the AgNPs in the denoted exposure environments, and incubated for 24 h. At the end of the exposure duration, ROS levels were measured via fluorescent analysis using a Synergy 4 BioTek microplate reader. Untreated, static A549 cells served as the negative control for normalization.

### 2.7. Fluorescent Evaluation of Stress Markers

A549 or A549/U937 cells were plated into 24-well plates at 2 × 10^5^ cells per well, returned to the incubator overnight, then treated with denoted experimental conditions. Following a 24 h exposure, the cells underwent fluorescent evaluation for heat shock protein 70 (HSP70) and actin. Cells were washed, fixed with 4% paraformaldehyde, permeabilized with 1% Triton X-100, and blocked with a bovine serum albumin solution. The fixed cells were then incubated with primary antibodies specific to either human HSP70 or actin (ThermoFisher), followed by incubation with the corresponding secondary antibody. The fluorescence intensity was measured using a Synergy 4 BioTek microplate reader (Winooski, VT, USA). Untreated, static A549 cells served as the negative control for normalization.

### 2.8. Cytokine Secretion Analysis

For these analyses, the cellular systems were set up as described above, with cells seeded into 24-well plates at 2 × 10^5^ cells per well, incubated overnight to return to homeostasis, and exposed to the denoted experimental conditions. After a 24 h exposure, the media was collected from the 24-well plate, and the unbound AgNPs were removed from solution via centrifugation. The supernatant, which contains secreted cytokines, underwent analysis for interleukin 1β (IL-1β), IL-6, IL-8, and tumor necrosis factor α (TNF- α) using protein specific ELISA kits from ThermoFisher, in accordance with the manufacturer’s recommendations. The same media source was used for all experimentation in order to remove the variability of different fetal bovine serum contents.

### 2.9. Data Analysis

All data are presented as the mean ± the standard error of the mean. For all experimentation, three independent trials were carried out in triplicate per trial. For all analyses, a two-way ANOVA with Bonferroni post-test was run using GraphPad Prism, with * indicating statistical significance from untreated, static A549 cultures and † indicating significance between the static and dynamic exposure for A549/U937 co-cultures.

## 3. Results

### 3.1. AgNP Characterization

**Figure 1:**
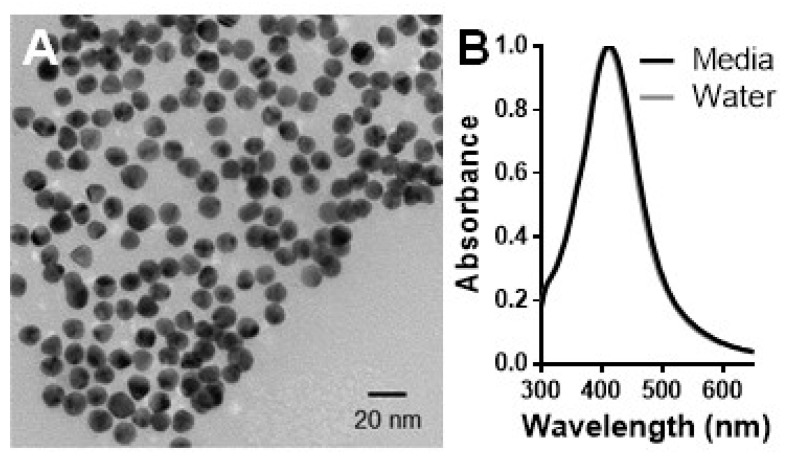
Characterization of the AgNP stock solution. (**A**) A representative TEM image verified the spherical morphology, uniform nature, and primary size of the experimental 10 nm citrate coated AgNPs. (**B**) The spectral signature of the AgNPs was visualized using UV–VIS in both water and complete media at a concentration of 25 µg/mL.

In this study, the effects of 10 nm, citrate coated AgNPs on enhanced in vitro systems were evaluated. Prior to cellular exposure, the experimental AgNPs underwent a standard array of characterization assessments [33]. Extensive material characterization was necessary as the unique physicochemical properties of each NP set are able to dictate both the nano-cellular interface and subsequent toxicological profile. As seen from TEM analysis (Figure 1A) the AgNPs were spherical in morphology and of uniform size distribution. Using multiple images the primary particle size of the experimental particles was found to be 11.2 ± 1.4 nm. Particle uniformity was further verified through UV–VIS analysis (Figure 1B), with the spectral signature displaying a single, sharp peak. 

As all nanoparticles will agglomerate to some degree in solution, which can impact their rates of sedimentation and cellular internalization, the extent of AgNP agglomeration was examined [34]. As seen in Table 1, the degree of agglomeration in both solutions was minimal. As expected, there was a slight increase in media due to the formation of a protein corona, which has been shown to increase the aggregate size of nanoparticles in solution [35]. This mild increase in aggregation was further confirmed by UV–VIS which displayed no significant spectral shift. In addition to agglomerate size, the zeta potential, or surface charge of the particles, was determined in both fluids. The approximate surface charges of −30 mV and −10 mV in water and media respectively are in agreement with the known charges of citrate and biological proteins, suggesting complete particle coating and a fully-developed protein corona [35,36]. Lastly, the degree of ionic dissolution has been linked to AgNP-dependent nanotoxicity, as silver ions have been shown to cause oxidative stress and intracellular damage [9,10]. As seen in Table 1, the rate of ionic dissolution in media was minimal. 

### 3.2. Establishment of In Vitro Exposure Environments

**Figure 2:**
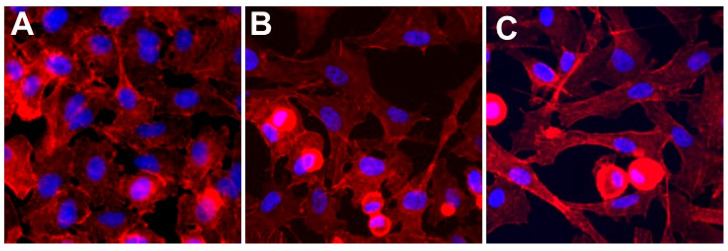
Experimental in vitro systems. In this study, three in vitro systems were utilized and include (**A**) the A549 human lung epithelial, (**B**) the A549/U937 epithelial/macrophage co-culture under static conditions, and (**C**) the co-culture model maintained within the presence of physiologically-representative fluid dynamics. In these representative images, actin and nuclei are shown as red and blue, respectively.

Historically, nanotoxicological studies have utilized a traditional in vitro model for investigation. While these cell systems have numerous advantages, they are not necessarily representative of a true exposure environment. In this study, a human lung epithelial, A549, model was employed, as well as an A549/U937 co-culture that incorporated an active macrophage line, providing an immune element. Moreover, the A549/U937 co-culture model was operated under both static and dynamic conditions, incorporating a second in vitro modification through the introduction of fluid flow. Fluorescence imaging was performed to visualize each of these experimental in vitro systems (Figure 2). Under static conditions, the A549s display traditional globular patterns, whereas the U937s are circular with dense actin. Within a dynamic environment, the A549 cells become elongated, which is more indicative of what is seen within in vivo models [37]. Moreover, these images confirmed that the co-culture was able to be successfully sustained under both static and dynamic conditions with no apparent loss of viability in the absence of AgNPs.

In order to state that any observed alterations to nanotoxicological profiles between static and dynamic conditions were due to cellular influences, it was necessary to verify that the presence of fluid dynamics did not alter AgNP characteristics. Therefore, AgNPs were circulated through the pump system, under acellular conditions, then characterized as before. As seen in Table 1, there were minimal alterations in the agglomerate size, zeta potential, and rate of ionic dissolution under dynamic conditions. These findings confirmed that the low shear rate impacted by the physiological fluid flow did not alter AgNP physicochemical characteristics. 

### 3.3. Evaluation of the Nano-Cellular Interface

**Figure 3:**
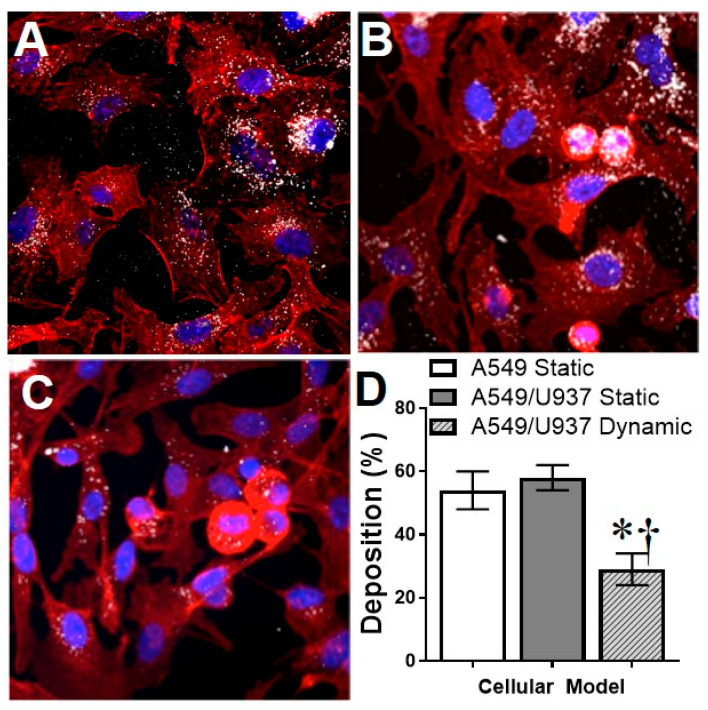
Evaluation of the AgNP-cellular interactions. Following a 24 h exposure to 5 µg/mL AgNPs the nano-cellular interface was visualized for the (**A**) static A549, (**B**) static A549/U937 co-culture, and (**C**) dynamic A549/U937 co-culture systems. In these images, actin and nuclei are stained red and blue, respectively with AgNPs appearing as white. (**D**) After a 24 h exposure, the AgNP deposition efficiency was measured via ICP-MS for all experimental systems. * and † denote statistical significance from untreated static A549 cells and between A549/U937 static and dynamic at the same AgNP concentration, respectively, *n* = 3, *p* < 0.05.

The extent of NP interactions with the surrounding cellular environment has been shown to be a predominant influence in the observed bioreponses post exposure [13,14]. In this study, an experimental AgNP exposure concentration of 5 µg/mL was selected, as it is low enough to induce intracellular stress without a corresponding loss of cell viability. Following a 24 h AgNP exposure the three cellular systems were visualized to qualitatively assess the nano-cellular interface (Figure 3). As seen in Figure 3A,B, there was extensive AgNP deposition onto the cellular surfaces. In the static co-culture AgNPs were found clustered within the macrophages, which was expected as the primary function of macrophages is to seek out foreign material [38]. In comparison, when examining the dynamic co-culture the quantity of AgNPs interacting with the cells appears to be significantly diminished, demonstrating that fluid flow could reshape the nano-cellular interface. 

In order to quantitatively determine the deposition within each in vitro model, the cellular systems were exposed to AgNPs for 24 h, washed to remove unbound particles, and then underwent ICP-MS analysis for Ag content. The static A549 and static co-culture models displayed equivalent deposition rates, approximately 55%. This was not surprising as particle sedimentation forces are the predominant transport mechanism in static systems [39]. In agreement with fluorescent imaging, when the co-culture was exposed to AgNPs under dynamic conditions, there was a significant drop in deposition efficiency, to an approximate 30% level.

### 3.4. Cellular Viability Analysis

**Figure 4:**
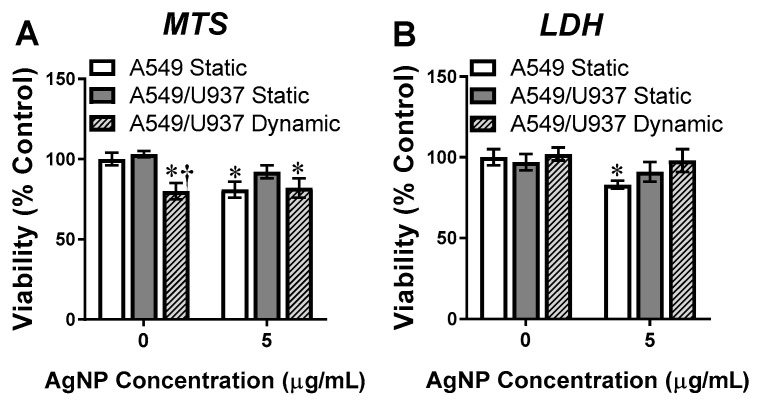
In vitro viability determination. The denoted cellular system was incubated for 24 h either without AgNPs or following a 5 µg/mL exposure. The in vitro models were then analyzed for cellular viability using either (**A**) MTS or (**B**) LDH assays. * and † denote statistical significance from static, untreated A549s and between A549/U937 static and dynamic at the same AgNP concentration, respectively, *n* = 3, *p* < 0.05.

Next, the cellular viability was determined for each in vitro system, both with and without AgNP exposure. This was done in order to verify that the presence of fluid dynamics was not inducing cytotoxicity and that the 5 µg/mL dosage was appropriate for future experiments. As seen in Figure 4, cell viability was determined using both MTS and LDH assessments. As these assays function through different mechanisms, examining two cytotoxicity endpoints will provide a more holistic evaluation [32]. Looking first at MTS results (Figure 4A), which monitors mitochondrial function of live cells, the dynamic flow was associated with a 20% reduction in viable cells versus the static co-culture, even in the absence of AgNPs. Following incubation with AgNPs, the static A549s displayed an approximate 20% cell death, as expected. The presence of immune cells did protect the A549s, with negligible toxicity identified in this model. Interestingly, there was no change in cell viability for the dynamic co-culture between the AgNP dosed and untreated cells, suggesting no NP-induced cytotoxicity. 

LDH results for AgNP-dependent cytotoxicity were slightly different from the MTS assay, as this method actively measures the degree of membrane disruptions which is directly correlated to cellular death (Figure 4B). Mainly, there was no loss of viability detected in the absence of AgNPs, for all experimental systems. Following a 24 h exposure to AgNPs, a 20% level of cytotoxicity was identified for static A549 cells, which is in agreement with MTS results. Regardless of flow condition, there was no substantial loss of viability detected for the A549/U937 co-culture. Taken together these results suggest that the apparent loss of viability associated with the dynamic co-culture in the MTS analysis may be an artifact in need of further exploration.

### 3.5. AgNP-Dependent Stress Varies with Cellular System

**Figure 5:**
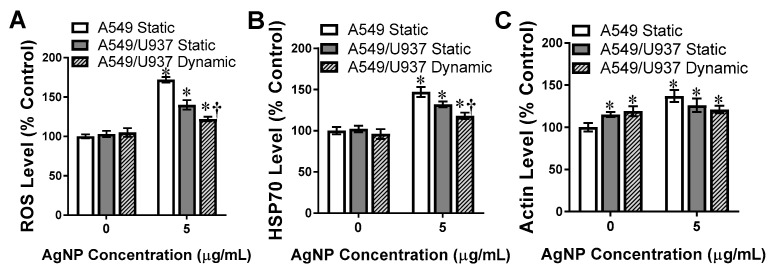
Activation of intracellular stress markers by AgNPs. The denoted experimental in vitro systems were incubated in either fresh media or a 5 µg/mL AgNP solution for 24 h, followed by evaluation of the stress markers (**A**) ROS, (**B**), HSP70, and (**C**) Actin inflammation. * and † denote statistical significance from static, untreated A549 cells and between A549/U937 static and dynamic at the same AgNP concentration, respectively, *n* = 3, *p* < 0.05.

AgNPs have been shown to induce numerous intracellular stress pathways as part of the dependent toxicity profiles. ROS production is a hallmark marker for intracellular stress as its activation is a known precursor for apoptosis, and is an upstream regulator of numerous stress and signaling cascades [40]. As seen in Figure 5A, basal ROS levels were equivalent for all in vitro models, demonstrating that dynamic flow did not stimulate a substantial stress response. Following AgNP exposure, the ROS level within A549 cells increased 70%, which is in agreement with the mild toxicity. In the static co-culture, this level was reduced as the macrophages were able to provide a level of protection and seek out AgNPs. Under dynamic conditions, the ROS levels were further reduced in the co-culture, with only a 20% increase, due to the reduced AgNP deposition in the presence of fluid flow.

The next stress marker evaluated was HSP70, which is known to be activated following AgNP exposure [41]. In the absence of AgNPs, all in vitro models displayed equivalent HSP70 levels (Figure 5B). Following AgNP incubation the HSP70 results mirrored ROS, with A549, static A549/U937, and dynamic A549/U937 associated with decreasing levels; even though they are elevated over untreated controls. There was a statistical difference between static and dynamic co-culture levels, indicating that fluid dynamics influenced the stress response to AgNPs.

When cells are experiencing significant stress the actin will become disorganized and inflamed, as a secondary stress response impacting cellular structure and functionality [42]. Prior to AgNP exposure, the A549/U937 co-culture was associated with a higher level of actin (Figure 5C). This is likely due to the greater fluorescence associated with the macrophages, as seen in Figure 2. Following AgNP exposure, clear actin inflammation was noted in the A549 system. However, regardless of flow condition, the actin expression in the A549/U937 co-culture was unchanged from untreated conditions. These results suggest that the stress response in the co-culture was not severe enough to trigger the actin inflammatory response in vitro.

### 3.6. Secretion of Pro-Inflammatory Cytokines

**Figure 6:**
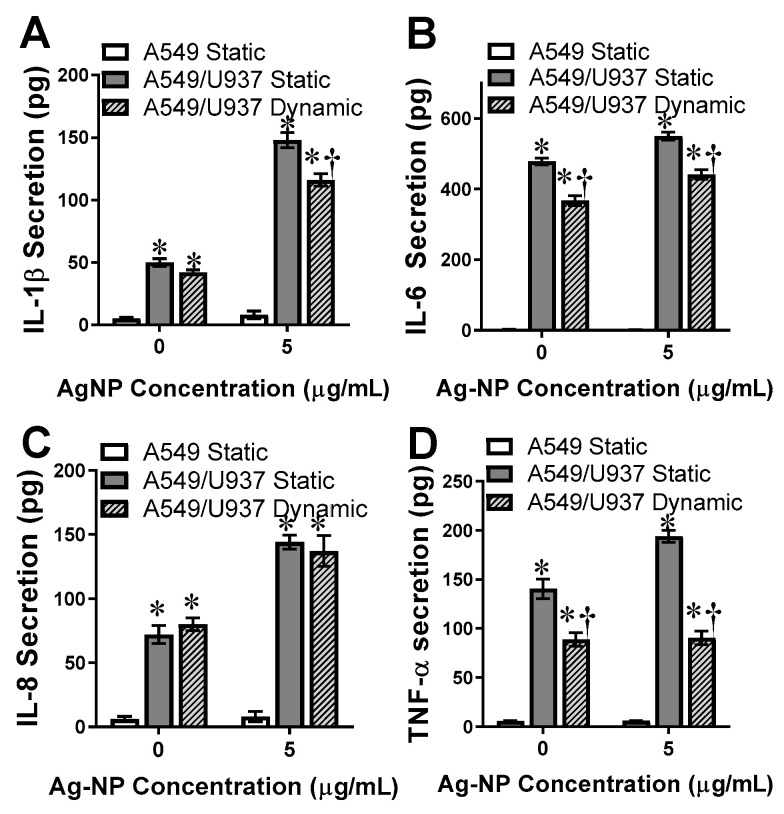
Secretion of pro-inflammatory cytokines as a marker of intracellular stress. The in vitro systems underwent a 24 h exposure, either untreated or following introduction of 5 µg/mL AgNPs, after which the media was evaluated for secreted levels of the pro-inflammatory cytokines of (**A**) IL-1β, (**B**), IL-6, (**C**) IL-8, and (**D**) TNF-α. * and † denote statistical significance from static, untreated A549 cells and between A549/U937 static and dynamic at the same AgNP concentration, respectively, *n* = 3, *p* < 0.05.

AgNP-dependent acute stress induction has been linked to the activation of inflammatory responses in vitro [43]. Once initiated, one early inflammatory response is the production and secretion of pro-inflammatory cytokines, including IL-1β, IL-6, IL-8, and TNF-α, in addition to the production of a spectrum of other cytokines and chemokines. As A549s are epithelial cells and not a significant player in the immune response, they are known to secrete insignificant cytokines quantities, which was verified by this study (Figure 6).

However, the addition of U937s to the in vitro model incorporated an immune element and resulted in a substantial inflammatory reaction. As seen in Figure 6, all four of the evaluated cytokines displayed significant production levels for both treated and untreated conditions. For Il-1β, IL-6, and TNF-α, AgNPs increased cytokine production and the presence of fluid dynamics was associated with lower secretion levels over static conditions. This decrease in cytokine production was in agreement with previously observed deposition and stress responses. IL-8 levels were elevated following AgNP exposure conditions, however, there was no difference noted between static and dynamic conditions. Taken together, these results demonstrated that cytokines production is dependent on numerous factors, including in vitro model composition, fluid dynamics, and the presence of an external stressor.

## 4. Discussion

As materials scientists and engineers have mastered the art of designing and synthesizing distinctive nanomaterials, their utilization in everyday products and applications has grown exponentially. The rise of nanotechnology has proven to be a double-edged sword, however, as the same physicochemical properties that make these materials attractive for applications also make them a hazard to living biological systems [44]. As each unique NP set has the potential to elicit differential toxicological profiles, this has created the vast need to evaluate the health and safety of every particle type. Given the challenges associated with in vivo experimentation, including the extended time frame, cost, and ethical concerns, the bulk of these initial nanotoxicological evaluations have been carried out in vitro, producing varied and contradictory results [45]. As such, one emerging goal is to develop relevant and robust model systems that can help bridge the gap in safety evaluations of experimental NPs.

This study implemented a previously established lung co-culture, consisting of human alveolar epithelial and activated macrophages, grown together in a 3:1 ratio [27]. This co-culture model was operated under both static and dynamic conditions, which allowed for the systematic evaluation of the influence of each in vitro modification. The first step was to verify the health and functionality of these experimental in vitro models. Looking at fluorescent images (Figure 2), the cells in all models appear healthy with no apparent signs of stress of loss of viability. There was a noticeable elongation for the dynamic model, aligning with the direction of fluid flow, which has been associated with other dynamic models [46]. When examining cell viability (Figure 4), the MTS results identified a 20% reduction in live co-culture cell numbers under dynamic conditions. However, LDH analysis determined there was no discernable cytotoxicity. As previous studies have reported that fluid flow was capable of reducing proliferation, without causing apoptosis, it is believed that this similar effect occurred in the dynamic A549/U937 co-culture [47,48].

Due to the functional differences associated with each experimental in vitro system, differential responses were observed following exposure to a low concentration of 10 nm AgNPs. Even though the deposition efficiencies were similar between the A549 mono-culture and the A549/U937 static co-culture (Figure 3), the presence of activated macrophages did reduce AgNP-dependent toxicity outcomes. Following exposure, A549s exhibited mild cytotoxicity and corresponding increases in ROS, HSP70, and actin inflammation. The inclusion of an immune cell line, which actively engulfed AgNPs [49], eliminated any cytotoxicity and significantly reduced intracellular stress levels (Figure 4 and Figure 5). This work is comparable to previous studies which identified differential response to AgNPs within co-culture models. For example, Zhang et al. reported that not only were AgNPs able to translocate through a human airway co-culture model but that the cells experienced a mild cytotoxic and inflammatory response; similar to the ones observed in this study [50]. In a second study, a Caco-2/THP-1 intestinal co-culture was found to more closely mimic the in vivo environment, with the addition of THP-1 cells minimizing the cytotoxic outcomes of AgNPs [51].

When examining the differences between the static and dynamic co-culture models several conclusions can be drawn. Firstly, the presence of lateral fluid movement appeared to be the predominant form of particle transport and was able to override the downward diffusion and sedimentation of the particles, with the result of a substantial drop in deposition. Previous studies have also observed similar trends with a decrease in NP internalization under shear flow in vitro [52]. This decreased deposition did translate to lower stress and inflammatory responses within the A549/U937 model. The most pronounced was the production of pro-inflammatory cytokines, which saw a substantial reduction under dynamic conditions. As cytokine production has the potential to induce systemic response through the activation of surrounding cells, this observed differential response could lead to significant changes within an in vivo system [53].

One long term goal of this work is to explore if enhanced in vitro systems, which retain the advantages of cell-based environments but incorporate key attributes of in vivo models, can be implemented for nanotoxicological evaluation. The design and utilization of these enhanced microenvironments affords the opportunity to explore new metrics for health and safety evaluations that could extrapolate to in vivo predictive modeling. The results presented in this study demonstrated that including active immune elements and dynamic fluid movement resulted in modifications to both the nano-cellular interface and subsequent biological outcomes over a standard in vitro control. As such, this study demonstrates the effectiveness of enhanced in vitro systems to more accurately elucidate NP-cellular interactions and improve the ability to accurately evaluate the safety of engineered nanomaterials.

## Figures and Tables

**Table 1:** AgNP characterization within multiple exposure environments.

	Primary Size (nm)	Agglomerate Size (nm)		Zeta Potential (mV)		Ionic Dissolution (%)
	11.2 ± 1.4	Water	Media	Water	Media	
Static		17.8 ± 0.9	27.0 ± 1.8	−30.2 ± 0.6	−9.3 ± 1.1	1.2 ± 0.4
Dynamic		18.3 ± 1.4	25.5 ± 2.6	−29.7 ± 0.7	−9.5 ± 1.3	1.4 ± 0.5
